# The Impact of Leadership and Management on the Implementation of Electronic Health Record Systems in the Primary Healthcare Centers

**DOI:** 10.3390/healthcare12202013

**Published:** 2024-10-10

**Authors:** Haitham Alzghaibi, Hayley A. Hutchings

**Affiliations:** 1 Department of Health Informatics, College of Applied Medical Sciences, Qassim University, Buraydah 51452, Saudi Arabia; 2 Medical School, Institute of Life Sciences 2, Swansea University, Singleton Park, Swansea SA2 8PP, UK; h.a.hutchings@swansea.ac.uk

**Keywords:** electronic health record, primary healthcare centers, centralized project management, large-scale projects, IT implementation, leadership, project management

## Abstract

Background: In the last three decades, Electronic Health Records Systems (EHRSs) have become one of the top priorities of policymakers globally. Nowadays, EHRS reform is fast becoming a priority in developed countries. The introduction of EHRSs in Saudi healthcare organizations is considered one of the highest priorities of policymakers. Saudi Arabian e-health strategy pays reasonable attention to the EHRS implementation project. According to Saudi Vision 2030, the e-health transformation will be on top of IT projects in the country. However, an estimated 50% of EHRS implementations have failed. Project leadership and type of project management have been found to be crucial components of effective EHRS implementation. Aim of the study: To evaluate the role of centralized project management (CPM) in the implementation of large-scale EHRSs in Primary Healthcare Centers (PHCs) in Saudi Arabia (SA). Methods: A sequential explanatory mixed-methods approach involving a survey and semi-structured qualitative interview methods were utilized. Results: A total of 39 (75%) out of 53 policymakers at the Saudi Ministry of Health completed the survey, and 14 project team members were interviewed. Findings from both illustrated that adopting centralized project management approaches to implementation was beneficial in facilitating large-scale EHRS implementation and helped to overcome barriers that may have otherwise led to the failure of the project. EHRS interoperability and software selection were the factors that CPM influenced most positively.

## 1. Introduction

Early research in Health Informatics (HI) has emphasized that the implementation of clinical computing should be a primary goal in order to enhance patient safety and care quality [1,2,3,4,5,6]. This idea came into existence in the early part of the 1960s and is still being proposed with modifications such as technological advancements. Such modifications will simplify the procedure so that patient care can be provided accurately and on time [7]. Nowadays, Electronic Health Record System (EHRS) reform is fast becoming a priority in developed countries [4,8,9]. Along with EHRS reform, the issue of improving patient safety and quality of care has justifiably been prioritized throughout the world by most healthcare givers [4,5,10]. Unfortunately, due to a variety of factors, EHRS reform has not received the same attention from the decision-makers in developing countries [11]. However, the introduction of EHRSs in Saudi healthcare organizations is considered one of the highest priorities of policymakers [4,12]. However, knowledge about the usefulness and benefits of EHRSs, as well as the likely implementation costs and other barriers, is considered scant in developing countries [13].

CPM refers to an organizational strategic approach in which project management activities are aggregated and coordinated by a specialized central unit or team [14,15]. At the core of a CPM organization lies a specialized unit predominantly comprised of project managers. Furthermore, this unit may encompass associated positions such as scrum masters, business analysts, or testers. The major objective of the company is to effectively manage and deliver projects in a timely manner [16,17]. Typically, individuals in leadership positions within CPM organizations possess substantial expertise and formal education in the field of project management [15,16].

Project leadership and management have been found to be crucial components of effective EHRS implementation [15,17,18,19,20,21,22]. Leadership and project management have been shown to have a positive impact on EHRS implementation and increase the possibility of successful implementation [22,23,24,25].

The project manager position can be occupied by a technical staff member, physician, or any manager within the organization [18]. The project manager should have the required skills, receive training in dealing with complex projects [18], and be capable of leading [26] and distributing workloads to project team members to encourage them to reach their goals [27]. Moreover, enabling long-term communication between project team members [26] and ensuring the involvement of EHRS end-users is integral to the lead project management role [25].

It has been suggested that such a management concept might not be capable of meeting the requirements of the population’s future healthcare plans and strategies [28]. However, it can become capable if the steps of implementation are well planned and there are clearly defined roles and responsibilities for lower-level management. A possible solution could be offering more control, authority, and power to the regional directorates at the health affairs in each region [28]. It is worth noting that according to Saudi Vision 2030, all 13 pre-defined health affairs are now converted to health clusters. Therefore, 13 health affairs are split into 30 health clusters [29]. According to Alghamdi and Urden [30], the Saudi MoH is leading and supervising its healthcare sectors in a purely centralized manner. It is responsible for formulating plans, legislation of regulations and policies, and managing and providing financial resources. Ekvall [31] argued that such CPM has several disadvantages, such as delaying projects, resistance to change, and a decrease in the level of innovation within the organization. This is thought to be due to the fact that the major decisions are being made by very few people [31]. However, a very recent study revealed that CPM contributes to the consistency of procedures and quality of any organization [32].

It has been suggested that such a management concept might not be capable of meeting the requirements of the population’s future healthcare plans and strategies [28]. However, it can become capable if the steps of implementation are well planned, separate multiple roles, and distribute authorities and powers to lower-level management. A possible solution can be offering more control and power to the regional directorates at the health affairs in each region [28]. According to Alghamdi and Urden [30], the Saudi MoH is leading and supervising its healthcare sector’s Primary Healthcare Centers (PHC) and hospitals in a purely centralized manner. It is responsible for formulating plans, legislation of regulations and policies, and managing and providing financial resources. 

## 2. Methods

This research was conducted at the headquarters of the Saudi Ministry of Health (MoH). The Saudi MoH manages and oversees healthcare organizations in Saudi Arabia. According to the latest statistics, more than 430,000 employees work for the Saudi MoH [33]. Semi-structured interviews and self-administrated questionnaires were used to collect the data for the current study. Therefore, the adopted method is referred to as explanatory sequential mixed methods [34]. The purpose of using sequential explanatory mixed methods was to seek further description and clarification of the impact of CPM from the same population. Therefore, this study was conducted in two phases; the quantitative study was conducted first and then analyzed. Thereafter, the qualitative study was designed and developed based on the analysis of the quantitative phase. This method was useful for seeking further details and in-depth and rich information about the implementation of EHRSs in the PHCs and the impact of CPM on the success of the project. Based on the adopted research design, the semi-structured interview guide was built based on the findings of phase one.

### 2.1. Phase One: Survey Questionnaire

The authors used a self-administered questionnaire comprised of nine items (or questions) to collect their quantitative data [35,36,37]. The questionnaire asked for specific details regarding individual perceptions of the impact of CPM. The respondent was required to answer the questions using a 7-point Likert scale response: Strongly disagree (1), Disagree (2), Somewhat disagree (3), No opinion (4), Somewhat agree (5), Agree (6), Strongly agree (7). The authors conducted a pilot study of the preliminary questionnaire prior to use in the study.

### 2.2. Phase Two: Semi-Structured Interviews

According to Miles and Gilbert [38], semi-structured interviews are useful for gathering comprehensive and in-depth information. Therefore, the authors decided to conduct semi-structured interviews due to their ability to provide a comprehensive understanding and explanation of the influence of CPM in the implementation of large-scale EHRSs. Although the role of CPM was assessed quantitatively, it was difficult to gain further information about certain implications of CPM on pre-defined factors that influence EHRS implementation, such as EHRS interoperability [39] or project planning and strategizing [40]. Therefore, semi-structured interviews provided additional and detailed information to allow a better understanding of previously examined aspects. This approach was previously termed sequential explanatory mixed methods [34]. Therefore, questions in the interview guide were driven by the analysis of the quantitative study.

Thirteen one-on-one interviews took place with the selected participants. The interviews were conducted over a two-month period. Interviews were scheduled for up to two hours each; however, no time limits were applied with regard to how long the interview could last. Participants were informed that they could withdraw from the interview at any time. The interviews were digitally recorded using an iPad and an iPhone. In addition, field notes were taken during the interview to avoid interruption when new questions emerged during the interview. This allowed for asking more questions to overcome the omissions and also to clarify any comments made.

### 2.3. Population and Sampling

Policymakers and other project team members were identified as the most appropriate individuals to provide an in-depth description of EHRS implementation. Hartzler and McCarty [41] suggested conducting one-on-one interviews with key informants from the project team to explore EHRS implementation. Silverman [42] suggested using purposive sampling for research that involves interviews, where a researcher gathers samples based on the requirements of the interview participants who are related to the research topic.

Therefore, the study population comprises all project team members directly or indirectly involved in implementing a large-scale EHRS project in Saudi PHCs. These consisted, for example, of heads of relevant departments (IT and PHC departments), senior managers, IT engineers, and technicians. This potential population of participants within the Saudi MoH has varying backgrounds and experience, departments, occupations, and genders. The target sample consisted of all project team members (*n* = 53). Out of the 53, only 31 participated and completed the questionnaire, indicating a response rate of 59%.

Identification of the study sample was conducted over two phases. Initially, I visited the relevant departments at the Saudi MoH (IT department and PHC department). I provided these departments with a copy of the ethical approval and facilitator letters obtained from the Saudi MoH to allow access to details of the potential study population. I requested a list of names and contact details of project team members who were involved in the EHRS implementation. The list contained only twenty-seven names. I spent time at the MoH to familiarize myself with the EHRS implementation project. During this time, I held informal meetings with some of the participants to ensure their appropriateness for the study and also asked them if there were other members of the project team who were not identified on the lists, in particular those from outside the selected departments. I collected demographic information from participants to ensure they appropriately represented the population. A total of fifty-three participants were selected due to their involvement, knowledge, expertise, and participation in EHRS implementation projects in PHCs in SA.

Although bias in purposive sampling is higher compared to probability sampling techniques, this study failed to determine the participants’ departments. Since the participants came from two main departments, namely IT and PHC departments, the determination of departments may assist in reducing bias. However, I physically collected the questionnaires in person, and I can confirm that individuals from both departments participated and returned the questionnaires.

The selection process for the targeted sample took place over a 4-week period, during which the researcher visited the headquarters of the Saudi Ministry of Health. Participant involvement in project implementation and knowledge they held about EHRS implementation in PHCs in SA were considered when selecting our survey sample. To reach the most appropriate participants for this study, non-probability, purposive, snowball sampling was used [34,43]. The survey questionnaire was distributed to all project team members over a 6-week period, beginning in the second week of August 2019.

Identification of the study sample was conducted over two phases. Initially, we visited the relevant departments at the Saudi MoH (IT department and PHC department). We requested a list of names and contact details of project team members who were involved in the EHRS implementation. The list contained only twenty-seven names. Secondly, the snowball sampling technique was used to determine if there were other members of the project team that were not identified on the lists, in particular those from outside the selected departments. For semi-structured interviews, we followed the same procedures adopted above. Therefore, a total of fifty-three participants met the selection criteria for the semi-structured interviews due to their involvement, knowledge, expertise, and participation in EHRS implementation projects in PHCs in SA, see Table 1. Between October and November 2019, eleven one-on-one interviews were conducted with the selected participants over a two-month period. Each interview was scheduled for up to two hours [44].

### 2.4. Data Analysis

#### 2.4.1. Quantitative

All data from the questionnaire were coded in numerical categories into IBM SPSS Statistics version 22.0 (IBM Corp., Armonk, NY, USA) [45]. Firstly, a Cronbach’s alpha test was used to examine the data collection reliability. Thereafter, an initial descriptive analysis of the questionnaire data was undertaken.

The number of participants responding to each Likert response and percentages are included in the descriptive statistics. These responses were used to calculate the total agreement percentage for each question. The total agreement percentage was used to determine which items within a given scale had the highest level of agreement. The total agreement percentage was calculated by summing all those responding positively to a specific question (i.e., somewhat agree, agree, or strongly agree). The total agreement percentage scores were then ranked in order based on the total agreement within the scale. This can assist with ranking those items that have the same median.

#### 2.4.2. Qualitative

The qualitative data collected in the semi-structured interviews were analyzed using deductive thematic analysis [34]. Deductive thematic analysis has been found to be more efficient for explanatory sequential mixed methods, as all generated key codes were driven by the analysis of the quantitative phase. Although the thematic analysis was very time-consuming, NVivo assisted in overcoming this issue. For instance, we were able to recall all highlighted quotes related to one code in just one click instead of searching through all the transcripts again to find the quote. Therefore, NVivo V10 (QSR International) was used to analyze the qualitative data thematically. NVivo helps to manage rich text by categorizing and organizing it rather than analyzing the text, as other quantitative programs do.

Once the interviews were finished, I converted the audio file into text format and then a Microsoft Word document. The recordings were transcribed verbatim to make the context of the response and the content of the information clear. Therefore, the process of data analysis began immediately once the interviews were transcribed and then translated into English (for all those interviews conducted in Arabic). All transcripts were Microsoft Word files which were imported to the NVivo software (version 14). After all the interviews were conducted and transcribed, I removed any data that could identify individuals. The transcribed data were then compared against the original audio recordings to ensure accuracy. Subsequently, I sent the Arabic transcripts to an official translation agency in SA to verify the accuracy of the translated data. Additionally, both the Arabic and English versions of the transcripts were reviewed by three professional translators. Finally, all transcripts were sent to an inter-rater experienced in thematic analysis for further review and observations.

## 3. Results

### 3.1. Survey Questionnaire

The reliability of the data collection instrument was assessed using the Cronbach’s Alpha test. The scales in this study demonstrated excellent reliability, with a score of 0.94. 

Initially, the findings show no significant differences in terms of participants’ demographics, such as gender, position, and involvement in the implementation project. Table 2 shows the percentage of male and female participation in the survey. It is evident that participation was male-dominant, with 80.6% of participants being male. Female participation was found to be only 16.1%. This reflects the actual proportion of females and males in the Saudi MoH, where the majority of the staff are male. 

More than half of the participants recorded their involvement in previous EHRS implementation projects. The participants in the survey were found to be from diverse professional roles. Assistants formed the highest number of participants, at twenty-one (67.7%). Only one deputy manager participated in this study, and three participants held other positions. Finally, out of thirty-one participants, twenty (64.5%) declared that they were directly involved in the process of implementation, and five (16.1%) declared that they aided the process through an indirect connection. Six (19.4%) participants did not declare the nature of their involvement [44].

The impact of CPM was explored with nine items. Table 3 illustrates the impact of CPM on EHRS implementation. Overall, there was extremely high agreement on all items (above 90%), with median scores being six or seven. Item 1) “*Overall impact is positive*” showed an agreement of 96.8% and a median score of seven, where participants strongly agree that CPM positively influences EHRS implementation in PHCs in SA. The lowest rank was given to item 9) “*Improve project team communication*” (90.4%), which is also considered very high with a median score of six [44].

### 3.2. Semi-Structured Interviews

The authors invited fifty-three members of the project team to participate in the semi-structured interviews. However, only 14 participants agreed to participate in the semi-structured interviews. The project team members who agreed to participate in this study were categorized into five different roles. Three were general managers (GM), one was a software developer (SD), three were heads of department (HD), two were deputy heads of department (DHD), and five were data analysts (DA). As mentioned previously, we analyzed all transcripts from the semi-structured interviews using NVivo software, which generated seven main themes, see Table 4.

Overall, the findings showed a consensus that CPM had a positive impact on the implementation of EHRSs in PHCs in SA. Some of the project team emphasized that CPM was best suited to the nature of the management system in Saudi Arabia. Some of the participants stated that this type of management system was appropriate in SA due to the similarities of the functions and business processes in the PHCs. It was commented by some that those decisions related to EHRS implementation should be made centrally by the Saudi MoH to enhance the success of the project [44].

“…particularly in SA, because the PHCs in SA are similar, are under the Ministry’s management and all have the same functions”

(DA 2)

“In the SA, in particular, it is necessary to use CPM to ensure project success”

(SD 1)

However, others suggested that the utilization of a semi-centralized project management system is more effective for implementing a large-scale EHRS. They argued that the adoption of this type of management represented a major challenge to the Saudi MoH and would be more effective if the MoH involved representatives from regional sub-departments in decision-making. Therefore, decisions regarding EHRS implementation could be made in coordination with representatives from lower levels, such as health affairs administrators in the regions or members of the PHCs.

“CPM is a challenge for the Ministry represented by the IT department due to the scale of the project, and therefore must involve sub-departments, health affairs in regions and also PHCs”

(HD 2)

### 3.3. The Impact of CPM on Decision-Making

All participants agreed that CPM has a positive impact on decision-making. For example:

“CPM’s role is very positive to handle large-scale projects while making significant decisions such as selecting the type of EHRS. In addition, CPM can reduce planning and implementation time for large-scale projects”

(GM 1)

“CPM has a very positive impact on decision-making”

(DA 3)

### 3.4. The Impact of CPM on Geographical Challenges

The geographical nature of SA is considered a challenge that may affect the success of EHRS implementation.

“Geographical nature of SA was a challenge to the MoH. Therefore, CPM assists the project team”

(HD2)

Nevertheless, there was some disagreement; the head of the department thought that CPM had a negative impact on geographical factors.

“I think the effect is negative. Because CPM limited the involvement of other members from different regions in SA who could give better insight about their regions, especially geographical challenges”

(HD3)

On the other hand, one of the DAs believed that CPM had no impact on geographical factors.

“CPM have no impact on this aspect where geographic challenges consider as technological which then can be overcome by developing appropriate infrastructure”

(DA 2)

### 3.5. The Impact of CPM on EHRS Interoperability

Interoperability was another measured factor found to be positively influenced by CPM. CPM facilitates communication among project teams to unify decision-making regarding a system that can be interoperable in the future. In addition, this contributes to making the process of communication between the systems more efficient and smoother and minimizes any issues related to future EHRS interoperability. Consequently, this matter will reflect positively on EHRS implementation.

“EHRS interoperability is crucial, it is highly affected by CPM and cannot succeed unless CPM is adopted especially in large-scale projects where different systems may selected and then implemented. We considered interoperable EHRS because the decision related to the interoperability and data exchange was made by one project team at the headquarter of the Saudi MOH. Therefore, communication among the project team was easy and effective for this purpose. So, it has a very positive impact”

(HD1)

### 3.6. The Impact of CPM on the Scale of the EHRS Implementation Project

There was agreement on the positive impact of CPM in overcoming challenges related to the scale of the project. A general manager believed that the CPM was *“Certainly positive”* (GM1). Other participants stated, for example:

“Implementing EHRS in very large number of PHCs which exceeded 2000 is not an easy mission due to the complexity of any project related to the IT. If all decisions and procedures related to project will not be made centrally by one project team, many challenges and conflicts may raise, and the project may fail. So defiantly CPM has a very positive effect”

(HD 3)

“The CPM system has a positive effect regarding the implementation of the EHRS in a large number of centers”

(SD 1)

Firmly believing that CPM had a positive impact on the number of PHCs, a proposal was made to divide the PHCs into zones or regions to overcome any issues related to the scale of the project and facilitate the implementation process.

“It has a positive impact. I would probably recommend dividing them into groups of different regions or zones”

(DA 3)

### 3.7. The Impact of CPM on Planning EHRS Implementation in PHCs in SA

The majority of the project team agreed that CPM had a positive impact on planning for the implementation of an EHRS. For instance, DA 1 said CPM had a “very positive effect”, and GM3 stated that the impact of CPM was “positive”. One of the general managers illustrated that CPM helped to avoid many of the technical and administrative problems that occurred during the planning phase by standardizing any decisions and opinions. In addition, CPM helped to reduce the costs of the project since the planning occurred centrally at the Saudi MoH.

“It is better to be centralized, because when the planning and entire implementation process is centralized the cost and time of the project will be reduced.”

(GM2)

On the other hand, one of the DAs believed that CPM had a negative impact on EHRS implementation planning.

“The CPM has negative impact on the planning phase due to the limited involvement of other parties and stakeholders such as members of PHCs or patients”

(DA 3)

### 3.8. The Impact of CPM on Software Selection

According to the participants’ responses, CPM had a positive impact on the software selection process. As illustrated by one of the general managers, “CPM is very positive, without a doubt” (GM1). In this context, software selection should be carried out centrally and under the supervision of the Saudi MoH.

“Such a decision regarding the software selection should be centralised at the top management of the Ministry to avoid any compatibility issue especially for large-scale project where multiple software may implement”

(HD 2)

Likewise, one of the department heads emphasized the importance of software selection being made centrally at the top level of the Saudi MoH. He believed that lower-level organizations, such as regional health affairs, do not have the ability to select or even examine an EHRS due to a lack of IT expertise.

“Before choosing a system, we must evaluate and review the EHRS and compare prices, system usability, system efficiency, etc. These things cannot be done in health centers where there are no experts in IT”

(HD 3)

On the other hand, due to previous experience in similar EHRS implementation projects, one of the DAs claims to have adopted the concept of semi-centralized project management to enhance software selection.

“From the experience of previous implementation projects, there were many notes from regions about the system, so health affairs in the regions should participate in system selection”

(DA 2)

### 3.9. The Impact of CPM on Team Communication Processes

There was consensus that CPM had a positive impact on team communication. For instance, HD 2 said, “CPM has very positive effect on communications among project team”. As illustrated by some of the participants, project team communications were made at the headquarters in the Saudi MoH in the form of meetings and workshops. All such meetings and workshops also involved the project team at different departments and different regions. This greatly facilitated and accelerated the decision-making process.

“…CPM is better to ensure faster and more effective communication between the project team.”

(DA 2)

However, others thought that CPM had no influence on the communications among the project team.

“…due to the development of telecommunications and all other media meetings and other form of communication can be made from distance and all stakeholders can be involved remotely via SKYPE or video conferences”

(GM 1)

### 3.10. The Impact of CPM on Project Team Selection

Team selection was also positively affected by CPM. According to one of the general managers, team selection was easier and more efficient when conducted centrally by the Saudi MoH. According to GM2, “CPM is the best” in terms of enhancing project selection, and DHD1 said, “It has a very positive impact”.

“…CPM dramatically facilitates this process because project team selection is easier and of more effective”

(GM 1)

## 4. Discussion

This study was undertaken to explore the influence of CPM on the implementation of large-scale EHRSs within PHCs in SA. Consistent with prior research, the findings indicate that CPM can significantly contribute to overcoming the challenges associated with large-scale EHRS implementation projects. These challenges often stem from issues related to project management and leadership, as identified in various studies [6,22,46,47]. However, it is also acknowledged that despite the potential benefits of CPM, some EHRS projects may not achieve success; a point echoed in the literature [22,46,47,48].

Based on the study findings, CPM could enhance the success of large-scale EHRS implementation projects in PHCs. These findings are in line with those by Safdari and Ghazisaeidi [49], who also stated that CPM can contribute to the success of EHRS implementation. Our findings and those by Safdari and Ghazisaeidi [49] are in disagreement with the findings by [31], who argued that CPM has some disadvantages, such as delaying projects, resistance to change, and a decrease in the level of innovation within the organization.

One of the most significant contributions of CPM, as identified in this study, is its impact on systems interoperability. Interoperability issues have been widely recognized as a critical challenge in EHRS implementation, with the potential to derail entire projects [50,51,52,53,54,55]. Consequently, systems interoperability tops the list of factors influenced positively by CPM [6]. Therefore, CPM, by providing a structured and centralized approach to project management, can serve as a lifeline for organizations struggling with interoperability issues, helping them to navigate and overcome these challenges effectively.

Software selection emerged as the second most positively impacted factor by CPM. The study’s findings underscore the importance of meticulous system selection to ensure that the chosen software meets organizational requirements and operates efficiently. This is supported by previous studies, which emphasize the significance of usability and functionality in software selection [3,24,54,56,57,58]. According to the decision-makers involved in this study, CPM played a crucial role in facilitating the software selection process, overcoming various difficulties, and ensuring that all pre-implementation measures—such as planning and decision-making—were conducted effectively. Moreover, the positive influence of CPM extends beyond the pre-implementation phase, continuing to support project management and leadership measures through the post-implementation phase.

Notwithstanding the benefits identified with CPM, the research indicates a possible transition towards semi-centralized project management, specifically for projects of significant magnitude. Implementing this strategy has the potential to increase stakeholder awareness, namely among end-users of EHRSs, and consequently enhance user satisfaction. The implications of the data suggest that although CPM presents significant advantages, adopting a semi-centralized strategy could perhaps give a more equitable resolution that can effectively cater to the requirements of central control while also accommodating the flexibility sought by diverse stakeholders.

However, the study faced several limitations, primarily during the data collection phase. The frequent changes at senior management and ministerial levels within the Saudi Ministry of Health (MoH) between 2014 and 2022—during which eight different ministers were appointed—impacted the study’s data collection efforts. These personnel changes led to challenges in obtaining consistent and reliable data, as many participants were newly appointed and lacked familiarity with the strategic implementation plans. Furthermore, the sample size for the semi-structured interviews was affected by the layoff of several project team members and policymakers who had been scheduled for interviews.

## 5. Conclusions

The findings of this study underscore the notable impact that CPM may have in effectively tackling the intricate obstacles linked to extensive projects, specifically in the realm of EHRS installation. CPM has demonstrated its efficacy in surmounting challenges such as software selection, guaranteeing interoperability, and enhancing communication among project teams. These elements play a critical role in large-scale projects, as successful execution requires the alignment of various components and stakeholders.

Notwithstanding these favorable results, the study also emphasizes the necessity for additional investigation in order to ascertain the optimal project management methodology for extensive EHRS implementations. Particularly, a more comprehensive examination of the distinctions between centralized and semi-centralized project management frameworks is necessary. This research would yield significant insights into the most effective management structure for facilitating the implementation of EHRS, particularly within a range of healthcare environments.

Furthermore, it is imperative to conduct comparative studies in order to evaluate the preparedness of PHCs in SA in relation to PHCs in other developing nations, particularly among the Arab Gulf Countries (AGCs). The Arabic Gulf Cooperation Countries (AGCs), which have cultural, economic, and infrastructural parallels with SA, provide a pertinent framework for comprehending the impact of varying levels of financial readiness and project management structures on the effectiveness of EHRS deployment.

It is recommended that future studies prioritize the examination of these comparative analyses in order to identify similarities and differences that might contribute to the development of optimal strategies throughout the region. Investigations pertaining to the effects of CPM and FR on the implementation of EHRSs within AGCs have the potential to generate significant findings that could be relevant to other developing countries sharing comparable features. The aforementioned contribution would not just enhance comprehension of EHRS deployment but also facilitate the formulation of solutions customized to the distinct requirements and limitations of healthcare systems in developing nations.

What was already known about the topic:

- Leadership and type of project management play significant roles in the success of EHRS implementation;
- Factors such as EHRSs end-user satisfaction and software selection are directly influenced by the project’s management and leadership.
- What this study added to our knowledge:
- CPM enhances the level of EHRS implementation success, especially in large-scale projects;
- Challenges such as EHRS interoperability can be overcome by the adoption of CPM;
- Guidelines to implement the EHRSs in multiple sites such as PHCs.

## Figures and Tables

**Table 1:** Sample selection criteria.

Inclusion	Exclusion
Project team member	EHRS end-users
Directly or indirectly involved in the implementation process	PHCs staff
Policymaker	
Administrative and clinicians who were part of the project team	

**Table 2:** Participant distribution based on gender, previous involvement in EHRS implementation projects, position, and role in the implementation project (*n* = 31).

Gender		
	No.	%
Male	25	80.6
Female	5	16.1
Missing	1	3.3
Previous involvement in EHRS implementation projects		
YES	18	58.1
NO	13	41.9
Position		
General Manager	3	9.7
Deputy Manager	1	3.2
Head of Department	3	9.7
Deputy Head of Department	3	9.7
Assistant	21	67.7
Role in the implementation project		
Direct	20	64.5
Indirect	5	16.1

**Table 3:** Participant responses to items representing the impact of CPM on EHRS implementation in PHCs.

	Items	Strongly Disagree	Disagree	Somewhat Disagree	No Opinion	Somewhat Agree	Agree	Strongly Agree	Median	Total Agreement	Rank
Overall impact is positive	N				1		5	25	7	30	1
	%				3.2		16.1	80.6		96.8	
Improve systems integration and interoperability	N				1		12	18	7	30	2
	%				3.2		38.7	58.1		96.8	
Better software selection	N		1		1	1	7	21	7	29	3
	%		3.2		3.2	3.2	22.6	67.7		93.5	
Easier to manage EHRS implementation in a large number of PHCs that are widely dispersed	N			1	1		12	17	7	29	4
	%			3.2	3.2		38.7	54.8		93.5	
Better project team selection	N				2	4	8	17	7	29	5
	%				6.5	12.9	25.8	54.8		93.5	
Better decision-making	N		1		2		6	22	7	28	6
	%		3.2		6.5		19.4	71.0		90.4	
Improve implementation of the strategic plan	N		1		2	1	9	18	7	28	7
	%		3.2		6.5	3.2	29.0	58.1		90.4	
Help leading and managing the project	N			1	1	3	10	15	6.50	28	8
	%			3.2	3.2	9.7	32.3	48.4		90.4	
Improve project team communication	N				3	1	18	9	6	28	9
	%				9.7	3.2	58.1	29.0		90.4	

**Table 4:** List of key codes.

Main Theme	Key Code
The impact of CPM on the implementation of the EHRS	The impact of CPM on decision-making
	The impact of CPM on geographical challenges
	The impact of CPM on EHRS interoperability
	The impact of CPM on the scale of the EHRS implementation project
	The impact of CPM on planning EHRS implementation in PHCs in SA
	The impact of CPM on team communication processes
	The impact of CPM on project team selection

## Data Availability

The datasets generated and/or analyzed in the current study are not publicly available due to copyright and ownership. All primary data collected for this research belong to the researchers. The dataset includes other data that will be used for another manuscript but are available from the corresponding author upon reasonable request.

## Abbreviations

MoH: Ministry of Health; EHRS: Electronic Health Record System; PHC: Primary Health Care; MoH: Ministry of Health; SA: Saudi Arabia; GM: General Manager; HD: Head of Department; DHD: Deputy Head of Department; SD: Software Developer; DA: Data Analyst; FR: Financial Recourses; SPSS: Statistical Package for the Social Sciences; CITIES: The Clinical Information System Implementation Evaluation Scale; GPs: General Practitioners.
